# The Study on the Relationship between Normalized Difference Vegetation Index and Fractional Green Canopy Cover in Five Selected Crops

**DOI:** 10.1155/2022/8479424

**Published:** 2022-03-21

**Authors:** Pavlo V. Lykhovyd, Raisa A. Vozhehova, Sergiy O. Lavrenko, Nataliya M. Lavrenko

**Affiliations:** ^1^Department of Marketing, Transfer of Innovations and Economic Studies, Institute of Irrigated Agriculture of NAAS, Kherson 73483, Ukraine; ^2^Institute of Irrigated Agriculture of NAAS, Kherson 73483, Ukraine; ^3^Department of Agriculture, Kherson State Agrarian and Economic University, Kherson 73006, Ukraine; ^4^Department of Land Management, Geodesy, and Cadaster, Kherson State Agrarian and Economic University, Kherson 73006, Ukraine

## Abstract

Crop models are of great use and importance in modern agriculture. Most models imply spatial vegetation indices, such as NDVI, or canopy cover characteristics, such as FGCC, to provide estimation of crops conditions and forecast productivity. The purpose of the study was to (1) determine the possibility of mutual conversion between spatial NDVI and Canopeo-derived FGCC in five crops (grain corn, sunflower, tomato, millet, and winter wheat) and (2) estimate the precision of such a conversion. The data set of the study was formed by the OneSoil AI derived satellite imagery on NDVI for the studied crops in different stages of their growing season combined with Canopeo-processed photographs of vegetating crops in the field with FGCC percentage calculation. The sets of NDVI and FGCC values were paired up and then statistically processed to obtain polynomial equations of NDVI into FGCC and inverse conversion for each crop. The results of the study revealed that mutual conversion between spatial NDVI and Canopeo-derived FGCC is possible. There is a strong direct correlation (*R*^2^ within 0.6779–0.9000 depending on the crop) between the studied indices for all crops. Close-growing crops, especially winter wheat, showed the highest correlation, while row crops and especially tomatoes had a less strong relationship between vegetation indices. The models for mutual conversion between FGCC and NDVI could be incorporated into the yield simulation models to improve the forecasting capacities.

## 1. Introduction

Normalized difference vegetation index (NDVI), developed and introduced by Rouse et al. [1], is the most used one to assess the conditions of vegetation cover both in agricultural and environmental monitoring purposes [2]. Even notwithstanding the fact that it is highly susceptible to atmospheric effects and soil background related distortions, it has become the most popular vegetation index in agricultural crop monitoring, which is mainly due to its simplicity and availability in “ready-to-use” state from most satellite and remote sensing data providers [3]. Applications of NDVI in precision agriculture systems embrace crop mapping, crop health monitoring, crop growth and development control, crop productivity estimation, etc. [2]. For example, crop producers can easily predict their yields in advance to harvesting period just using the average field NDVI values and simple gradual scales or models that is of great importance for crop production sector of the economy [4]. Therefore, most farmers are longing to have access to NDVI data. However, until now there is a great number of crop producers in Ukraine, who cannot afford paid services providing readily available NDVI with interpretation, while free-of-charge platforms often are not as easy in use, require specific knowledge to calculate the index from raw satellite imagery and interpret it correctly, while some land arrays are just absolutely missed in free open access services. Therefore, it is necessary to find an alternative solution to get NDVI for every Ukrainian field. One of the solutions could be the derivation of NDVI using some other indices, which every farmer could easily obtain in field conditions without specific knowledge, tools, and extra payments. One of such indices is fractional green canopy cover (FGCC), which became accessible for every smartphone user owing to the development of Canopeo mobile app [5]. Canopy cover, if properly screened with accordance to simple guidance, has been proved to be not inferior to NDVI in crop modelling [6], while other studies have found strong agreement between the values of FGCC and NDVI [3, 7]. At the same time, significant variability in the relationship between these two indices has also been proved for different crops [3, 8]. Therefore, it is necessary to develop “NDVI–FGCC” correlation models for every particular crop to convert FGCC into NDVI, which could be further utilised for agricultural monitoring purposes.

At the same time, sometimes it is required to perform an inverse conversion of NDVI into FGCC, which could also be of great use for some specific purposes in cases when FGCC is impossible to be directly measured using improvised means (this is mainly true for the areas with high vegetation, orchards, forests, bushes, etc.) [9–11]. In addition, FGCC values are also used as inputs in some models related to crop yield prediction [12, 13] and monitoring of natural flora objects [14]. Therefore, the model for the derivation of FGCC from NDVI is also of great importance for modern agricultural science and practice.

The aim of this study was to establish the relationship between NDVI and FGCC in five selected crops to provide the models for mutual conversion between both vegetation indices.

## 2. Materials and Methods

The study was conducted in 2021 with five selected crops: winter wheat, grain corn, millet, sunflower, and tomato. The relationship between NDVI and FGCC was established using the method of polynomial regression analysis of the gathered field (for FGCC) and satellite-based (for NDVI) data; 100 data pairs “NDVI–FGCC” were involved for each studied crop to create conversion polynomial regression models [15].

The crops, involved in the study, were located at the fields as follows: winter wheat, Kherson neighbourhood private farm, geographical coordinates of the field are 46.64° N 32.54° E (all the coordinates are given in decimal degrees); grain corn, a research field of the Institute of Irrigated Agriculture of NAAS, geographical coordinates of the field are 46.74° N 32.71° E; millet, a research field of the Institute of Irrigated Agriculture of NAAS, geographical coordinates of the field are 46.74° N 32.71° E; sunflower, a research field of the Institute of Irrigated Agriculture of NAAS, geographical coordinates of the field are 46.74° N 32.70° E; tomato, several research fields located at private farms of Kherson oblast with geographical coordinates 46.26°N 32.44°E, 46.29°N 32.01°E, 46.14°N 32.70°E, 46.57°N 32.38°E, 46.74°N 32.04°E, and 46.75°N 32.52°E.

The determination of FGCC and NDVI was carried out during the growing season of the crops studied at different stages of their development to embrace a higher diversity of plant conditions, namely, winter wheat, stem elongation, earing, milk ripening; grain corn, 3–5 leaves, 8–10 leaves, tasselling; millet, tillering, jointing, grain filling; sunflower, stem elongation and flower bud development, flowering, ripening; tomato, establishment of young plant, flowering, first fruit ripening.

The Canopeo mobile app was used to record the FGCC values in the selected fixed spots of the fields. Measurements were carried out strictly in accordance with the guidelines provided on the official app website https://canopeoapp.com/. For each crop, we collected 100 FGCC records. Considering the possible impact of the smartphone camera on the FGCC estimation results [16], we should mention that the Sony Xperia XZ2 Premium camera was used to take the vegetation screens.

The data on the corresponding NDVI values for each fixed spot on the research fields were collected from the service OneSoil AI (https://onesoil.ai/en/), which utilises Sentinel-1 satellite imagery to provide readily available NDVI screens for the selected fields with the pixel resolution of 5 × 5 m. NDVI values were taken for the same time period as FGCC images with a maximum delay of ±3 days. Then, the corresponding FGCC and NDVI values formed pairs for further statistical data processing in Microsoft Excel 365 package and BioStat v7 add-in [17].

## 3. Results and Discussion

### 3.1. Fractional Green Canopy Cover Conversion into Normalized Difference Vegetation Index

The results of regression analysis using polynomial function of the second grade testify that there is a strong intercorrelation between the values of FGCC and NDVI in every crop studied. The regression statistics for each crop are presented in Table 1, while the models for FGCC into NDVI conversion are provided in Table 2. The evidence is that the strongest connection between the indices studied is observed for winter wheat (coefficient of determination is 0.9000), while the slightest connection is recorded for tomato (coefficient of determination is 0.7182). This fact could be put on the peculiarities of tomato leafage and its general architectonic nature: It was the only crop in the study with a stem, which crawls around the ground. Besides, it is evident that row crops (tomato, sunflower, and grain corn) had less strong relationship between the studied indices than the close-growing crops (millet and winter wheat). This fact could be explained by a significant presence of soil in the spatial imagery for row crops. Therefore, the conversion of FGCC into NDVI will have higher accuracy and reliability for close-growing crops with little spacing (no more than 15 cm) spacing between rows.

**Figure 1:**
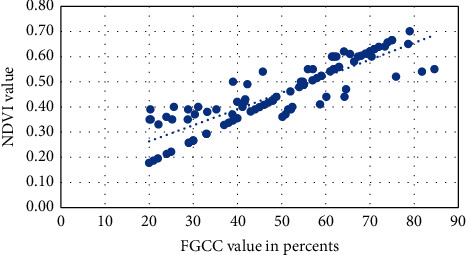
Approximation of the polynomial model for FGCC into NDVI conversion for grain corn.

**Figure 2:**
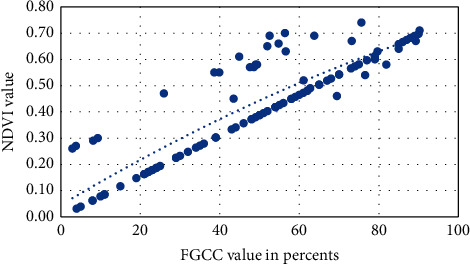
Approximation of the polynomial model for FGCC into NDVI conversion for sunflower.

**Figure 3:**
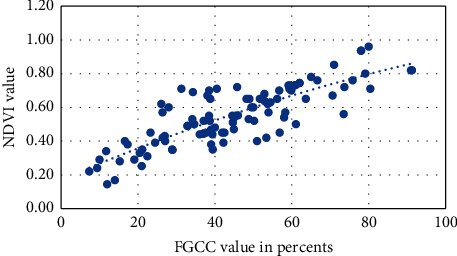
Approximation of the polynomial model for FGCC into NDVI conversion for tomato.

**Figure 4:**
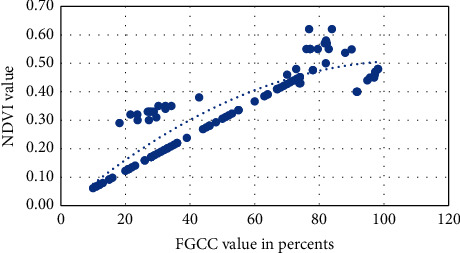
Approximation of the polynomial model for FGCC into NDVI conversion for millet.

**Figure 5:**
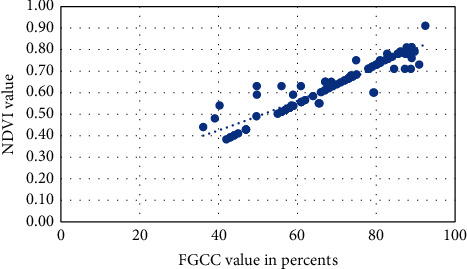
Approximation of the polynomial model for FGCC into NDVI conversion for winter wheat.

Figures 1–5 present visualization of the approximation of the conversion polynomial models FGCC to NDVI for each crop studied.

The results of previously conducted studies on the subject also show a significant high correlation between the measured FGCC and NDVI values in wheat crops [18]. Strong linear relationship between canopy cover and NDVI with the coefficient of determination *R*^2^ averaged to 0.96 has been proved for several vegetable crops in the study by Johnson and Trout [19]. Trout et al. [20] also support the statement mentioned above of strong mutual correlation between the FGCC and NDVI in the main horticultural crops. The study by Prabhakara et al. [21] has also claimed about strong linear relationship between the green canopy cover percentage and NDVI values, reaching the values of the coefficient of determination of 0.93 for particular cases. Reed et al. [12] pointed out that the FGCC and NDVI measurements of the Canopeo app in winter wheat crops are strongly correlated with the *R*^2^ value of 0.76. Therefore, our findings add some new information on the connection between directly measured FGCC and spatial NDVI for some major cereal and industrial crops and are in absolute agreement with the results of previous studies conducted abroad of Ukraine.

### 3.2. Normalized Difference Vegetation Index Conversion into Fractional Green Canopy Cover

At the same time, in some cases, it might be needed to convert NDVI values into FGCC. For example, FGCC is preferred to NDVI in studies when crop biomass productivity is estimated [22, 23]. Therefore, the conversion of NDVI to FGCC should also be provided.

The results of our study outline that the best accuracy in such a conversion is observed for winter wheat crops, while the lowest accuracy is again attributed to tomato (Table 3). Generally, the NDVI conversion into the FGCC conversion is slightly less accurate than the FGCC conversion into the NDVI for winter wheat, grain corn, and tomato, while it is more accurate for millet and sunflower. The reason for such a regulation is difficult to trace, but this is supposed to be mainly due to the algorithm of statistical processing of the data.

**Figure 6:**
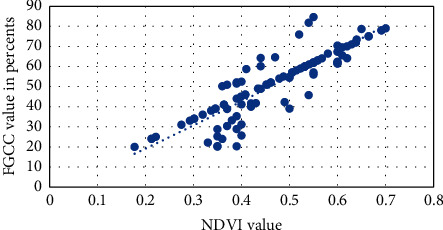
Approximation of the polynomial model for NDVI into FGCC conversion for grain corn.

**Figure 7:**
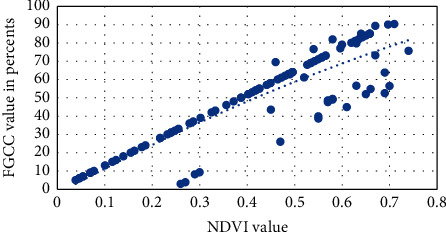
Approximation of the polynomial model for NDVI into FGCC conversion for sunflower.

**Figure 8:**
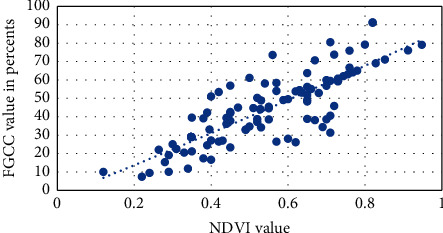
Approximation of the polynomial model for NDVI into FGCC conversion for tomato.

**Figure 9:**
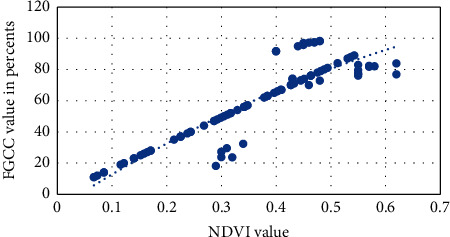
Approximation of the polynomial model for NDVI into FGCC conversion for millet.

**Figure 10:**
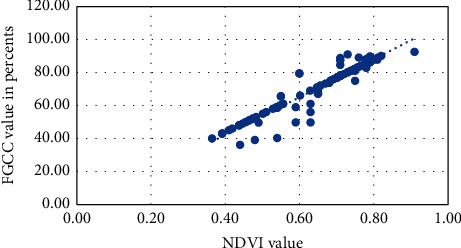
Approximation of the polynomial model for NDVI into FGCC conversion for winter wheat.

Visual approximation of the NDVI into FGCC polynomial models for each studied crop is presented in Figures 6–10. The models are given in Table 4.

The models for mutual conversion between FGCC and NDVI in the crops studied require further calibration and rigorous field tests for adjustment and enhancement of their predictive performance so that they could be trustworthy enough to be enrolled in the yield simulation models.

## 4. Conclusions

Mutual conversion between spatial NDVI and Canopeo-derived FGCC is possible. There is a strong direct correlation (*R*^2^ within 0.6779–0.9000 depending on the crop) between the studied indices for all crops. Close-growing crops, especially winter wheat, showed the highest correlation, while row crops and especially tomatoes had a less strong relationship between vegetation indices. In fact, if we consider row crops, we have a significant presence of soil in the remotely detected images. In this regard, several researchers recommend using the soil-adjusted vegetation index (SAVI), which belongs to the group of spatial vegetation indices with the least distortion connected with the soil properties and its presence on the remotely sensed imagery, thus providing better identification of plants and their discrimination from the soil [24]. This is a prospective direction for further investigations in this direction, which is quite promising for the enhancement of models' quality, as well as the increase in the number of crops enrolled in the study. The model “FGCC–NDVI” could be useful when remote sensing data are not available for some reasons, but it is possible to get a smartphone-based estimate of canopy cover percentage in Canopeo app, while the inverse conversion model could be applied to estimate canopy cover in the crops, which are difficult to be screened using the smartphone app (fruit trees, bushes, forests, etc.) but are provided with satellite imagery.

## Figures and Tables

**Table 1:** Regression statistics for the FGCC model developed into NDVI conversion for the crops studied.

Statistical index	Grain corn	Sunflower	Tomatoes	Millet	Winter wheat
Correlation coefficient *R*	0.8985	0.8795	0.8475	0.8887	0.9487
Coefficient of determination *R*^2^	0.8073	0.7735	0.7182	0.7899	0.9000
Adjusted *R*^2^	0.8033	0.7689	0.7124	0.7855	0.8979

**Table 2:** FGCC model in NDVI conversion for the crops studied.

Crop name	Conversion model
Grain corn	NDVI = 3 × 10^−6^×(FGCC) ^2^ + 0.0062 × FGCC + 0.1384
Sunflower	NDVI = −2 × 10^−5^×(FGCC)^2^ + 0.0091 × FGCC + 0.0448
Tomatoes	NDVI = −2 × 10^−5^×(FGCC)^2^ + 0.0098 × FGCC + 0.1718
Millet	NDVI = −5 × 10^−5^×(FGCC)^2^ + 0.0098 × FGCC + 0.0168
Winter wheat	NDVI = 2 × 10^−5^×(FGCC)^2^ + 0.0045 × FGCC + 0.2080

**Table 3:** Regression statistics for the developed models of NDVI into FGCC conversion for the studied crops.

Statistical index	Grain corn	Sunflower	Tomatoes	Millet	Winter wheat
Correlation coefficient *R*	0.8969	0.9000	0.8234	0.8927	0.9428
Coefficient of determination *R*^2^	0.8044	0.8099	0.6779	0.7969	0.8889
Adjusted *R*^2^	0.8004	0.8060	0.6713	0.7927	0.8866

**Table 4:** The NDVI models into the FGCC conversion for the studied crops.

Crop name	Conversion model
Grain corn	FGCC = 44.5291 × (NDVI)^2^ + 79.6233 × NDVI + 3.5721
Sunflower	FGCC = −19.1083 × (NDVI)^2^ + 127.2628 × NDVI − 0.7472
Tomatoes	FGCC = 19.0338 × (NDVI)^2^ + 70.0017 × NDVI + 0.0754
Millet	FGCC = −13.9308 × (NDVI)^2^ + 127.4283 × NDVI − 1.4352
Winter wheat	FGCC = −1.5687 × (NDVI)^2^ + 116.3299 × NDVI − 4.0062

## Data Availability

The raw data and photographs used in the study could be produced on reasonable request to the authors.
